# Evolutionary New Genes in a Growing Paradigm

**DOI:** 10.3390/genes13091605

**Published:** 2022-09-08

**Authors:** Esther Betrán, Manyuan Long

**Affiliations:** 1 Department of Biology, University of Texas at Arlington, Arlington, TX 76019, USA; 2 Department of Ecology & Evolution, The University of Chicago, Chicago, IL 60637, USA

How new genes evolve has become an interesting problem in biology, particularly in evolutionary biology. Without understanding the evolution of new genes, it would be difficult to understand many of the biological properties of organisms, as their complexity has been accumulating through evolutionary time. Without adequate knowledge about the evolutionary process of the origination of new genes, it would be impossible to understand the genetic bases of changes in gene functions and phenotypes as targets of natural selection and other evolutionary forces. The advancement of genomics and functional analyses of genes has made it feasible to investigate new gene evolution and data has been accumulating.

The 14 articles in this Special Issue on new gene evolution provide exciting evidence and discussion about this topic from several angles of study. They illustrate the scientific issues with respect to the evolution of new genes. They show that new genes exist widely in organisms and that their origination and evolution is one of the most common evolutionary processes [1,2]. In addition to providing new evidence that has accumulated on them and insights into the phenomenology of new gene origination, these contributions also present new and interesting scientific problems and concepts for further pursuits. They explore new dimensions of the evolution of new genes and re-examine old dimensions with new approaches, keeping the problem of new genes a growing field and preventing it from becoming idle or dead, something that scientists try hard to avoid [3]. 

It is now clear that some of the patterns of the origination of new genes have been consistent across the tree of life. For example, Li et al. [4] used comparative genomics to identify new genes in the starry flounder and found, consistently with previous observations, that DNA-mediated duplications are 10 times more abundant than either RNA-mediated duplications or the genes categorized as potentially de novo genes. Interestingly, in this work, they observed that some of the newly evolved genes were differentially expressed between the left and the right side of the starry flounder body, and their contribution to the asymmetric body plan of flatfishes needs to be explored from this new angle in the evolution of this developmental trait.

It has also been shown consistently that many new genes are expressed during spermatogenesis [5,6,7] and that the X chromosome might not be a good location for new testis-biased genes [8] but the Y chromosome is. While there might be multiple reasons for these two patterns, they likely involve the selection for male germline functions [9,10]. Some of the patterns are particularly strong for RNA-mediated duplicates or retrogenes [11]. Retrogenes studied in the mosquito in this issue of *Genes* [12] reveal that as sex chromosomes evolve, the patterns of retroduplication and expression change. In flies, genes with mitochondrial function have been duplicated and now have a sperm function, and one of them is studied in this issue of *Genes* [13]. Su et al. [14] analyzed single-cell transcriptomes and revealed complementary patterns of expression between new genes and parental genes revealing strong selection for those new genes. The evolution of the Y chromosome [15] has also been at the receiving end of innumerable gene duplications, as this is a good location for male-specific genes as long as purifying selection is efficient enough. 

In a computational analysis, Guo et al. [16] evaluated the role of frameshift mutations in the evolution of new gene functions after gene duplication, following up on a statement on page 80 of Ohno’s ground-breaking book *Evolution by Gene Duplication* that even if the chances of frameshift mutations of generating new functions are small, it might happen [17]. As Ohno acknowledges in other sections of the book, frameshift mutations are usually deleterious and are not observed in protein-coding genes, but Guo and colleagues reveal multiple examples of this in multiple genomes. These are remarkable examples of the extensive number of changes that sometimes occur in a particular region as a new gene with a new function originates. As a striking example in line with the argument that many changes can quickly take place in the genome as a new gene with a new function is evolving, Krinsky et al. [18] study the male germline transcriptional rewiring that can take place when a new gene evolves from a duplication of a transcription factor.

In an organism that contributed to the understanding of the origination of multicellularity, Luna and Chain [19] reported an exciting discovery from analyses of five dictyostelid species genomes, i.e., 24% of genes in the genomes are lineage- or species-specific. Further analyses indicate that the biased new gene duplicates, expressed in a particular developmental stage, show greater divergence in expression among orthologues and paralogues. The expression analyses also provided new data to support a pattern previously reported in other organisms: new genes show narrower expression patterns across developmental stages or tissues the younger they are, for example, *Drosophila* [6,20], *Oryza* [21], and primates [22].

Cancer-cell proliferation provides a short-term genomic evolutionary process in which the role of duplication at various levels, from genomic fragments to genes to whole-genome and fusions between genes, can be readily examined. Glenfield and Innan [23] provide an extensive and critical review of this area from genetic mechanisms to tumorigenesis to cancer evolution to therapeutic response. In particular, based on the contributions of the authors in bioinformatics and computational biology, the review of the bioinformatic tools for the identification of gene fusions from genomic sequences provides rich and valuable information for audiences who need to use these tools.

Antifreeze glycoproteins (AFGPs) were the first-ever known proteins created by de novo origination, i.e., genes that evolve from regions that were initially non-coding [24,25]. Zhuang and Cheng [26] provided an updated picture of how AFGPs in codfish species evolved their protein functions and their natural history with molecular evolution leading to gene family expansion.

Also related to the potential for the evolution of de novo genes, Lee et al. [27], in this issue of *Genes*, analyzed the microproteins in mammals and discussed their relevance to the evolution of new gene, in particular, to a hypothesis that these short genes may be formed from noncoding sequences. Genomes seem to be full of short peptides or short proteins (e.g., microproteins with <100 amino acids). Amazing observations were made that show obvious conservation among distant species, suggesting a selective constraint to maintain these microproteins with their functionality. This work adds to the clear evidence reported for de novo origination leading to two microproteins in the human genome and other primate species, evidence that was viewed to be difficult to find [28].

In addition, Grandchamp et al. [22], in this *Genes* volume, thoroughly examined four properties in human protogenes, i.e., the genes an in early stage of de novo origination [29]: intron acquisition, regulatory elements, UTRs, and domain evolution. The extensive data were characterized as showing significant differences between protogenes and old genes, revealing a growth process of gene structures with age. 

Another important aspect to understand de novo gene origination is the role of random peptides: Are random peptides relevant to biology and evolution? The answers to this problem have been largely negative since early experiments to test the potential functionality of random peptides [30,31]. From large libraries they previously published, Bhave and Tautz [32] detected surprisingly that higher than 10% of random peptides might have advantageous fitness effects on the growth of *E. coli*, and these peptides likely developed interactions with cellular proteins in various pathways. Castro and Tautz [33] further reported their unexpected finding of a structural preference of shorter peptides. These observations also reveal that the genomes of *E. coli* do not function perfectly. Therefore, despite their long-term evolution under natural selection, the artificially created random peptides were able to perform a positive fitness effect.

In essence, these contributions reveal previously unknown phenomena and processes and provide further in-depth analysis of recently detected properties with evolutionary new genes. The data and analyses presented in these articles have also unveiled fresh insights into a number of basic biological problems in understanding sex, gametogenesis, development, protein properties, genome structure, carcinogenesis, and multicellularity. We invite audiences to tour a world of increasing knowledge of how new genes originate in a growing paradigm in biology and evolution.

## References

[1] Long M., Betran E., Thornton K., Wang W. (2003). The origin of new genes: Glimpses from the young and old. Nat. Rev. Genet..

[2] Long M., VanKuren N.W., Chen S., Vibranovski M.D. (2013). New gene evolution: Little did we know. Annu. Rev. Genet..

[3] Kuhn T.S. (1962). The Structure of Scientific Revolution.

[4] Li H., Chen C., Wang Z., Wang K., Li Y., Wang W. (2021). Pattern of New Gene Origination in a Special Fish Lineage, the Flatfishes. Genes.

[5] Kaessmann H. (2010). Origins, evolution, and phenotypic impact of new genes. Genome Res..

[6] Zhang Y.E., Vibranovski M.D., Krinsky B.H., Long M. (2010). Age-dependent chromosomal distribution of male-biased genes in Drosophila. Genome Res..

[7] Assis R., Bachtrog D. (2013). Neofunctionalization of young duplicate genes in Drosophila. Proc. Natl. Acad. Sci. USA.

[8] Vibranovski M.D., Zhang Y., Long M. (2009). General gene movement off the X chromosome in the Drosophila genus. Genome Res..

[9] Kleene K.C. (2005). Sexual selection, genetic conflict, selfish genes, and the atypical patterns of gene expression in spermatogenic cells. Dev. Biol..

[10] Gallach M., Domingues S., Betran E. (2011). Gene duplication and the genome distribution of sex-biased genes. Int. J. Evol. Biol..

[11] Kaessmann H., Vinckenbosch N., Long M. (2009). RNA-based gene duplication: Mechanistic and evolutionary insights. Nat. Rev. Genet..

[12] Miller D., Chen J., Liang J., Betran E., Long M., Sharakhov I.V. (2022). Retrogene Duplication and Expression Patterns Shaped by the Evolution of Sex Chromosomes in Malaria Mosquitoes. Genes.

[13] Eslamieh M., Mirsalehi A., Markova D.N., Betran E. (2022). COX4-like, a Nuclear-Encoded Mitochondrial Gene Duplicate, Is Essential for Male Fertility in Drosophila melanogaster. Genes.

[14] Su Q., He H., Zhou Q. (2021). On the Origin and Evolution of Drosophila New Genes during Spermatogenesis. Genes.

[15] Ricchio J., Uno F., Carvalho A.B. (2021). New Genes in the Drosophila Y Chromosome: Lessons from *D. willistoni*. Genes.

[16] Guo B., Zou M., Sakamoto T., Innan H. (2022). Functional Innovation through Gene Duplication Followed by Frameshift Mutation. Genes.

[17] Ohno S. (1970). Evolution by Gene Duplication.

[18] Krinsky B.H., Arthur R.K., Xia S., Sosa D., Arsala D., White K.P., Long M. (2021). Rapid Cis-Trans Coevolution Driven by a Novel Gene Retroposed from a Eukaryotic Conserved CCR4-NOT Component in Drosophila. Genes.

[19] Luna S.K., Chain F.J.J. (2021). Lineage-Specific Genes and Family Expansions in Dictyostelid Genomes Display Expression Bias and Evolutionary Diversification during Development. Genes.

[20] Dai H., Yoshimatsu T.F., Long M. (2006). Retrogene movement within- and between-chromosomes in the evolution of Drosophila genomes. Gene.

[21] Zhang L., Ren Y., Yang T., Li G., Chen J., Gschwend A.R., Yu Y., Hou G., Zi J., Zhou R. (2019). Rapid evolution of protein diversity by de novo origination in Oryza. Nat. Ecol. Evol..

[22] Grandchamp A., Berk K., Dohmen E., Bornberg-Bauer E. (2022). New Genomic Signals Underlying the Emergence of Human Proto-Genes. Genes.

[23] Glenfield C., Innan H. (2021). Gene Duplication and Gene Fusion Are Important Drivers of Tumourigenesis during Cancer Evolution. Genes.

[24] Cheng C.-H.C., Zhuang X., Ramløv H., Friis D. (2020). Molecular Origins and Mechanisms of Fish Antifreeze Evolution. Antifreeze Proteins.

[25] Chen L., DeVries A.L., Cheng C.H. (1997). Evolution of antifreeze glycoprotein gene from a trypsinogen gene in Antarctic notothenioid fish. Proc. Natl. Acad. Sci. USA.

[26] Zhuang X., Cheng C.C. (2021). Propagation of a De Novo Gene under Natural Selection: Antifreeze Glycoprotein Genes and Their Evolutionary History in Codfishes. Genes.

[27] Lee J., Wacholder A., Carvunis A.R. (2021). Evolutionary Characterization of the Short Protein SPAAR. Genes.

[28] Levy A. (2019). How evolution builds genes from scratch. Nature.

[29] Carvunis A.R., Rolland T., Wapinski I., Calderwood M.A., Yildirim M.A., Simonis N., Charloteaux B., Hidalgo C.A., Barbette J., Santhanam B. (2012). Proto-genes and de novo gene birth. Nature.

[30] Davidson A.R., Sauer R.T. (1994). Folded proteins occur frequently in libraries of random amino acid sequences. Proc. Natl. Acad. Sci. USA.

[31] Keefe A.D., Szostak J.W. (2001). Functional proteins from a random-sequence library. Nature.

[32] Bhave D., Tautz D. (2021). Effects of the Expression of Random Sequence Clones on Growth and Transcriptome Regulation in Escherichia coli. Genes.

[33] Castro J.F., Tautz D. (2021). The Effects of Sequence Length and Composition of Random Sequence Peptides on the Growth of E. coli Cells. Genes.

